# A comprehensive in silico analysis, distribution and frequency of human *Nkx2-5* mutations; A critical gene in congenital heart disease

**DOI:** 10.15171/jcvtr.2019.47

**Published:** 2019-10-31

**Authors:** Samira Kalayinia, Serwa Ghasemi, Nejat Mahdieh

**Affiliations:** ^1^Cardiogenetic Research Center, Rajaie Cardiovascular Medical and Research Center, Iran University of Medical Sciences, Tehran, Iran; ^2^Department of Biology, School of Basic Sciences, Islamic Azad University Research Tehran Branch, Tehran, Iran

**Keywords:** Congenital Heart Disease, *Nkx2-5*, Mutation, Computational Analysis

## Abstract

***Introduction:*** Congenital heart disease (CHD) affects 1% to 2 % of live births. The *Nkx2-5* gene, is known as the significant heart marker during embryonic evolution and it is also necessary for the survival of cardiomyocytes and homeostasis in adulthood. In this study, *Nkx2-5* mutations are investigated to identify the frequency, distribution, functional consequences of mutations by using computational tools.

*** Methods:*** A complete literature search was conducted to find *Nkx2-5* mutations using the following key words: *Nkx2-5* and/or CHD and mutations. The mutations were in silico analyzed using tools which predict the pathogenicity of the variants. A picture of *Nkx2-5* protein and functional or structural effects of its variants were also figured using I-TASSER and STRING.

***Results:*** A total number of 105 mutations from 18 countries were introduced. The most (24.1%) and the least (1.49%) frequency of *Nkx2-5* mutations were observed in Europe and Africa, respectively. The c.73C>T and c.533C>T mutations are distributed worldwide. c.325G>T (62.5%) and c.896A>G (52.9%) had the most frequency. The most numbers of *Nkx2-5* mutations were reported from Germany. The c.541C>T had the highest CADD score (Phred score = 38) and the least was for c.380C>A (Phred score=0.002). 41.9% of mutations were predicted as potentially pathogenic by all prediction tools.

***Conclusion:*** This is the first report of the *Nkx2-5* mutations evaluation in the worldwide. Given that the high frequency of mutation in Germany, and also some mutations were seen only in this country, therefore, presumably the main origin of *Nkx2-5* mutations arise from Germany.

## Introduction

Congenital heart disease (CHD) is the most common defect in heart structure^1^ that occurring 1%-2% of live births and 10% of abortions.^2,3^ In spite of numerous research studies aiming to detect CHD reasons, the exact etiology of this disease is still obscure. The past decades studies estimated that chromosomal abnormalities and single gene disorders result in 8% of CHDs.^1^

Some transcriptional factors regulate cardiac development including GATA binding protein 4 (*GATA4*), T-box transcription factor (*TBX*) and NK2 home box 5 (*Nkx2-5*). These are as CHD prime causes, with topping the list of *Nkx2-5.* ^4^ The *Nkx2-5* gene, a highly conserved gene from Drosophila to humans, is located on chromosome 5q35 and contained two exons. It is known as the significant heart marker during embryonic evolution and it is also necessary for the survival of cardiomyocytes and homeostasis in adulthood. The most of *Nkx2-5* mutations have been observed in CHD cases, including tetralogy of Fallot (TOF), ventricular septal defect (VSD), atrial septal defect (ASD), and transposition of the great arteries (TGA).^5^ Studies in human and animal models indicated Nkx2-5 expression only in cardiac tissue, thereby emphasizing its significant role in heart development. Mice which lacked even one copy of *Nkx2-5* gene, represented various heart abnormalities. Furthermore, it has been observed that *Nkx2-5* is involved in postnatal heart protection.^1^

Any changes in the genes, especially critical genes in a specific pathway, have a significant effect on the health. Evaluation of *Nkx2-5* mutations can prepare early diagnosis of a variety of CHDs. These mutations display dominant inheritance pattern. Mutation screening can also determine family members that may be at risk. To our knowledge, there is no comprehensive study about frequencies and distribution of *Nkx2-5* mutations in the worldwide populations. In this study, we reviewed all mutations of *Nkx2-5* which reported up to now.

## Materials and Methods

### Searching methods and data collection

The FASTA format of *Nkx2-5* reference genome and protein sequence was downloaded from the UCSC database (build37/hg 19 version). As a first attempt to investigate the relationships between *Nkx2-5* mutations and CHD, the search was performed in the database of PubMed, Google Scholar, John Wiley, and Elsevier. Some key words were applied in our literature search such as: “CHD”, “*Nkx2-5”* and “mutation”. Searching the literature was without any limitation in language and time. For finding any other reported mutations, we surveyed in the several free public databases for genetic variations, such as the single nucleotide polymorphism database (dbSNP),^6^ the human gene mutation database (HGMD),^7^ the exome aggregation consortium (ExAC)^8^ and 1000 Genome.^9^ All the statistical analyses were performed using IBM SPSS Statistics for Windows, version 24.0 (Armonk, NY: IBM Corp).

### In silico analyses

The functional and pathogenicity consensus of mutations were predicted using computational tools such as Mutation Taster,^10^ Sorting Intolerant From Tolerant, version 6.2.1 (SIFT),^11^ Polymorphism Phenotyping, version 1.03 (Polyphen2),^12^ PROVEAN, version 2.0.23^13^ and combined annotation dependent depletion (CADD), version 1.3.^14^ SIFT interprets results using the TrEMBL (version 34.3) and Swiss-Prot (version 51.3) and classifies mutations as deleterious (<0.05) and/or tolerated (≥0.05).^11^ PolyPhen2 predicts the impact of an amino acid change on protein structure and function by applying protein 3D structure and multiple sequence alignment. It classifies mutations as possibly damaging, probably damaging, or benign.^12^ The PROVEAN also classifies mutations as deleterious or natural. These tools evaluate the functional consequences of mutations at five principal levels; protein stability, posttranslational, translational, transcriptional, and splicing. The protein FASTA sequence (NP_004378.1) of selected mutations was used as the input file of these prediction tools.

### Protein structure prediction

I-TASSER (Iterative Threading Assembly Refinement) was applied for evaluating the Nkx2-5 protein structure/function resulting from the mutation with the most frequency. It is a platform which generates some models of query protein applying state-of-art algorithms. The quality of protein features prediction, is judged with some scores; C-score (confidence score), models with C-score >- 1.5 have correct fold; TM-score (template modeling score), the value range is in [0, 1],^15^ a higher score displays a better structure; and RMSD, with the range similar to TM-score, determines accuracy of the model. I-TASSER also predicts solvent accessibility with the range from 9 (highly exposed) to 0 (buried) residue position.^16^

### Protein network prediction

The STRING database version 10.5^17^ was used for describing of the proteins which have interaction with Nkx-2.5 protein. This databaseprovides a useful evaluation of protein-protein associations, including physical and functional interactions.

## Results

### Literature review

**Figure 1:**
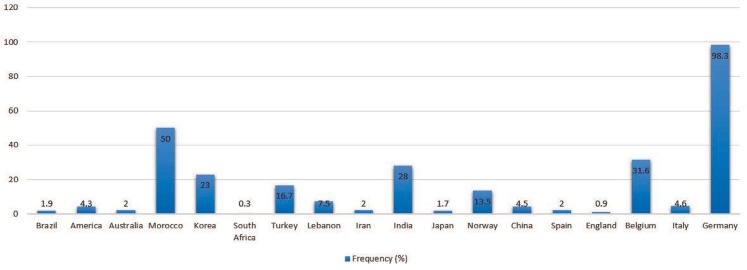
Frequency of *Nkx2-5* mutations in some countries.

**Figure 2:**
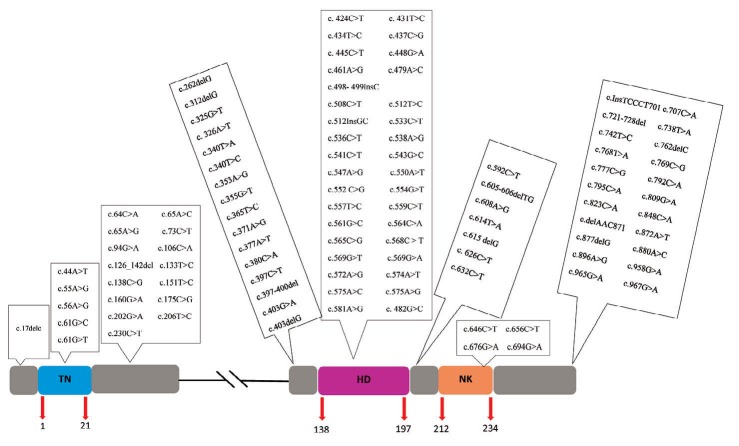
The location distribution of reported *NKX2.5* mutations. The broken line shows the single intron. UTR, untranslated region; NK, conserved domain; HD, home domain; TN, conserved domain. Gray boxes are out site of the coding region.

A total number of 59 articles were surveyed. We found 105 mutations (Table 1) containing 80 missenses, 12 deletions, 3 insertions, and 10 nonsenses. These mutations were documented from 18 countries, which among them, America, Germany and China had the most number of *Nkx2-5* mutations, respectively (Figure 1). We obtained some significance information from these searches including: mutation features according to DNA and protein sequences, CHD type related to any mutation, the number of reported affected cases harboring specific mutation, the total number of studied individuals, and the place where study performed there. The number of studied individuals were as 2230, 1199, 2827, 335 and 146 individuals in America, Europe, Asia, Africa and Australia, respectively. 30 mutations in America (c.533C>T with the most frequency), 49 in Europe (c.896A>G with most frequency), 46 in Asia (c.738T>A with most frequency) and 2 mutations both in Africa and Australia have been found. Moreover, the frequency of *Nkx2-5* mutations was as 4.12% in America, 24.1% in Europe, 6.15% in Asia, 1.5% in Africa and 2% in Australia. The location of *Nkx2-5* mutations was illustrated in Figure 2.

**Table 1:** The reported of *Nkx2-5* mutations

**Mutation**		**Mutation Type**	**America** ^15, 18-29^	**Europe** ^25, 28, 30-44^	**Asia** ^1, 29, 45-67^	**Africa** ^68,69^	**Australia** ^70^	**CHD Type**	**A/B**
**DNA**	**Protein**								
c.17delC	p.A6V	Indel			1/235			TOF	1/235
c.44A>T	P.K15I	Missense	1/608					SASD	1/608
c.55A>G	p.N19D	Missense			2/136			AF,AVB	2/136
c.56A>G	P.N19S	Missense		18/68				VSD	18/68
c.61G>C	p.E21Q	Missense	1/608		2/146		1/146	TOF,AF	4/720
c.61G>T	p.E21ter	Nonsense		1/28				AVSD,HLH	1/28
c.64C>A	p.Q22K	Missense			1/268			ASD	1/268
c.65A>C	p.Q22P	Missense	1/608					ASD	1/608
c.65A>G	p.Q22R	Missense	1/28		2/100			TOF,VSD	3/128
c.73C>T	p.R25C	Missense	10/987	7/466	21/311			TOF,HLHS,TA,IAA, AVC,TD.DCM,VSD,ASD,PFO	38/1764
c.94G>A	p.E32K	Missense			1/13			ASD	1/13
c.106C>A	p.R36S	Missense			1/268			VSD	1/268
c.126_142del	p.P43Gf59	Indel			1/58			ASD	1/58
c.133T>C	p.S45P	Missense		1/68				VSD	1/68
c.138C>G	p.C46W	Missense			1/58			ASD	1/58
c.151T>C	p.F51L	Missense		1/68				VSD	1/68
c.160G>A	p.E54K	Missense			1/268			TOF	1/268
c.175C>G	p.P59A	Missense			3/136			VSD	3/136
c.202G>A	p.E68K	Missense			1/13			ASD	1/13
c.206T>C	p.L69P	Missense		1/68				VSD	1/68
c.230C>T	P.P77L	Missense		1/68				VSD	1/68
c.262delG	p.A88Xfs	Indel			1/18			ASD	1/18
c.312delG	p.K104fs	Indel		2/12				AVB,ASD	2/12
c.325G>T	p.E109ter	Nonsense		5/8				ASD,VSD,PAS,PFO,AVB	5/8
c. 326A>T	p.E109V	Missense			1/140			ASD	1/140
c.340T>A	P.C114S	Missense		5/68				ASD,AVSD	5/68
c.340T>C	p.C114R	Missense		22/68				AVSD	22/68
c.353A>G	p.K118R	Missense						ASD,VSD	5/68
c.355G>T	p.A119S	Missense	1/220	3/169				TD,DCM,AVSD,HLHS	4/389
c.365T>C	P.L122P	Missense		6/399				ASD	1/331
c.371A>G	p.K124R	Missense		6/68				ASD	6/68
c.377A>T	p.E126V	Missense		11/68				ASD,VSD,AVSD	11/68
c.380C>A	p.A127E	Missense	1/608					SASD	1/608
c.397C>T	p.P133S	Missense		6/68				ASD	6/68
c.397-400del	p.P133Gfster42	Indel			1/3			Hetrotaxi	1/3
c.403G>A	p.A135T	Missense		9/68				VSD,AVSD	9/68
c.403delG	p.A135Rfst	Indel			11/185			ASD,VSD,PDA,TOF,AF	11/185
c. 424C>T	p.R142C	Missense		13/50				ASD,VSD,TOF	13/50
c. 431T>C	p.L144T	Missense		11/68				VSD,AVSD	11/68
c.434T>C	p.F145S	Missense	1/160		1/110			FA,AF	2/270
c.437C>G	p.S146W	Missense			1/260			DCM,AVB,AF	1/260
c. 445C>T	p.Q149ter	Nonsense	3/92	7/52	1/26			ASD,VSD,AVB	11/170
c.448G>A	p.V150I	Missense		1/7				VSD	1/7
c.461A>G	p.E154G	Missense			2/213			ASD,AVB	2/213
c.479A>C	p.Q160P	Missense				4/8		ASD.AVB	4/8
c. 482G>C	P.R161P	Missense		2/141				TD	2/141
c.498- 499ins C	p.E167fs	Indel		1/16				ASD,VSD,AVB	1/16
c.508C>T	p.Q170ter	Nonsense	4/33					ASD,AVB	4/33
c.512T>C	p.L171P	Missense	9/48					ASD,VSD,AVB	9/48
c.512InsGC	p.A172Rfs	Indel	3/8					ASD	3/8
c.533C>T	p.T178M	Missense	12/33		1/18		2/146	ASD,AVB	15/197
c.536C>T	p.S179F	Missense			5/226			ASD	5/226
c.538A>G	p.T180A	Missense			2/146			AF	2/146
c.541C>T	p.Q181ter	Nonsense		1/331				ASD	1/331
c.543G>C	P.Q81H	Missense	4/11					ASD,AVSD,VSD	4/11
c.547A>G	p.K183E	Missense		29/68				VSD,AVSD	29/68
c.550A>T	p.I184F	Missense	1/23					DCM,ASD,VSD,AVB	1/23
c.552 C>G	p.I184M	Missense	2/245					DCM,ASD,VSD,AVB	2/245
c.554G>T	p.W185L	Missense		3/16				ASD,VSD,AVB	3/16
c.557T>C	p.F186S	Missense			2/136			AF,AVB	2/136
c.559C>T	p.Q187ter	Nonsense		6/68				ASD	6/68
c.561G>C	p.Q187H	Missense		2/50				ASD,VSD,TOF	2/50
c.564C>A	p.N188K	Missense	5/92		1/26			ASD,VSD,AVB	5/118
c.565C>G	p.R189G	Missense	5/92		1/26			ASD,VSD,AVB	6/118
c.568C > T	p.R190C	Missense			1/18			ASD	1/18
c.569G>T	p.R190L	Missense		2/121				AVB	2/121
c.569G>A	p.R190H	Missense	3/48	2/121				ASD,VSD,AVB	5/169
c.572A>G	p.Y191C	Missense	1/92		1/26			ASD,VSD,AVB	2/118
c.574A>T	p.K192X	Nonsense			5/142			BAV	5/142
c.575A>C	p.K192T	Missense		6/68				ASD	6/68
c.575A>G	p.K192R	Missense		2/68				ASD	2/68
c.581A>G	p.L194R	Missense		2/68				ASD	2/68
c.592C>T	p.Q198ter	Nonsense	3/33		2/98			AVB,ASD	5/131
c.605- 606delTG	p.L202fs	Indel		3/16				ASD,VSD,AVB	3/16
c.608A>G	p.E203G	Missense			1/150			VSD	1/150
c.614T>A	p.V205E	Missense		6/68				ASD	6/68
c.615delG	p.L207Cfs	Indel			2/213			ASD,AVB	2/213
c. 626C>T	p.P209L	Missense			1 /140			VSD	1/140
c.632C>T	P.P211L	Missense		2/50				ASD,VSD,TOF	2/50
c.646C>T	p.R216C	Missense	1/608		38/150			ASD,VSD,PDH,COA,TOF,PHT	39/758
c.656C>T	p.A219V	Missense	1/608					TOF,ASD	1/608
c.676G>A	p.D226N	Missense			3/150			VSD,ASD,PDH	3/150
c.694G>A	p.G232R	Missense		1/331				ASD	1/331
c.InsTCCCT701	P.A235ApFSter	Indel	1/608	5/68				SASD,AVB,ASD	6/676
c.707C>A	p.P236H	Missense				1/327		ICA	1/327
c.721-728del	p.Y241fs	Indel			2/213			ASD,AVB	2/213
c.738T>A	p.N246K	Missense			42/185			ASD,VSD,PDA,TOF,AF	42/185
c.742T>C	p.Y248H	Missense		5/68				ASD	5/68
c.762delC	p.A255Pfs	Indel		1/121				ASD,AVB	1/121
c.768T>A	p.Y256ter	Missense		1/7				ASD,AVB	1/7
c.769C>G	p.P257A	Missense			1/30			VSD	1/30
c.777C>G	p.Y259ter	Nonsense	7/92		1/26			ASD,VSD, AVB	8/118
c.792C>A	p.C264ter	Nonsense			1/109			ASD	1/109
c.795C>A	p.S265R	Missense		3/8				TD	3/8
c.809G>A	p.C270Y	Missense			2/213			ASD,AVB	2/213
c.823C>A	p. P275T	Missense	1/608					COA	1/608
c.848C>A	p.P283Q	Missense			1/135			VSD, PDA, AS	1/135
c.delAAC871	p.del291N	Indel	1/608					DORV	1/608
c.872A>T	p.N291	Missense			1/100			TD,AF	1/100
c.877delG	p.V293ter	Indel	4/15					WP	4/15
c.880A>C	p.N294H	Missense		14/68				AVSD	14/68
c.896A>G	p.D299G	Missense		36/68				ASD,VSD,AVSD	36/68
c.958G>A	p.G320S	Missense		17/68				ASD,AVSD,VSD	17/68
c.965G>A	p.R322Q	Missense		2/68				ASD	2/68
c.967G>A	p.A323T	Missense	1/608					TOF	1/608
The number of mutations in studied patients			30	49	46	2	2		
The number of studied individuals in studied patients			2230	1199	2827	335	146		
The number of patients carrying mutation in studied patients			92	289	174	5	3		
The frequency of mutation in studied patients			4.12	24.10	6.15	1.49	2.05		

TGA, Transposition of the Great Arteries; DORV, Double Outlet Right Ventricle; TOF, Tetralogy of Fallot; TAPVR, Total Anomalous Pulmonary Venous Return; TAPVC ,Total Anomalous Pulmonary Venous Connection; PAPVC, Partial Anomalous Pulmonary Venous Connection; ASD, Atrial Septal Defect; AVSD, Atrioventricular septal defect; VSD, Ventricular Septal Defect; HLH, Hypoplastic Left Heart; PS, Pulmonary Stenosis; PAS, Pulmonary artery stenosis; AS, Aortic stenosis; SVAS, supravalvar aortic stenosis; COA, Coarctation of the aorta; PDA, Patent Ductus Arteriosus; BAV, Bicuspid aortic valve. PFO: ( patent foramen ovale); PHT: ( Pulmonary hypertension); LSVC; (left superior vena cava ); SASD: (secundum atrial septal defect); IAA: (Interrupted aortic arch ); PHT: (Pulmonary hypertension); CCHD: ( Critical congenital heart disease ); WPW: (Wolff-Parkinson-White);AVC:(AV connection ); PLSVC: (Persistent left superior vena cava ); WP: (Wenckebach periodicity)A: the number of patients carrying mutation; B: The number of studied individuals.

### Frequency and distribution of the mutations

**Figure 3:**
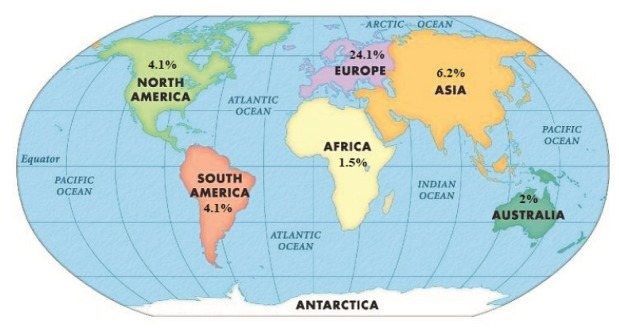
The frequency of the Nkx2-5 mutations in continents. Frequency percentage Nkx2-5 mutations was shown the below of the continent name.

The c.73C>T was detected in 8 countries including: America, Spain, Brazil, Italy, Germany, Korea, Lebanon and Turkey. The c.533C>T was also observed in 4 countries like America, Germany, Japan and Australia. These findings indicate that the distribution of c.73C>T and c.533C>T mutations are more than other *Nkx2-5* mutations in worldwide. c.325G>T (62.5%) and c.896A>G (52.9%) had the most frequency, although the distribution of them was only in Germany. The most numbers of *Nkx2-5* mutations were reported from Germany, among them, c.896A>G (94.5%) and c.547A>G (42.6%) were the most common mutations in this country. The frequency of the *Nkx2-5* mutations in continents were indicated in Figure 3.

### Bioinformatics

Computational analyses of the 105 mutations, predicted pathogenic effect for most of them (Table 2). SIFT tool predictions were included: 55 deleterious, 38 tolerable and 12 not available (N/A). Polyphen2 determined 27 possibility damaging, 37 probably damaging, 29 benign and 12 N/A. Using PROVEAN also detected 53 deleterious, 40 natural and 12 N/A. The c.541C>T had the highest CADD score (Phred score=38) and the least was for c.380C>A (Phred score=0.002).

**Table 2:** *In Silico* analyses of *Nkx2-5* mutations

**Mutation**	**Mutation type**	**Frequency**	**dbSNP**	**HGMD**	**Mutation taster**	**Polyphen2**	**PROVEAN**	**SIFT**	**CADD**	**EXAC**	**1000G**
c.17delc	Indel	0.42 %	_	CM123285	DC	BENIGN	NE	DE	25.4	_	_
c.44A>T	Missense	0.16%	387906773	CM033925	DC	PRD	DE	DE	28.9	_	_
c.55A>G	Missense	1.47%	_	_	DC	POD	NE	TO	24.3	_	_
c.56A>G	Missense	26/47%	_	_	DC	POD	NE	TO	22.9	_	_
c.61G>C	Missense	0.55%	104893904	CM013525	DC	PRD	NE	DE	26.4	91	_
c.61G>T	Nonsense	3.57%	104893904	CM013525	DC	NA	NA	NA	37	_	_
c.64C>A	Missense	0.37%	764389026	CM1110282	DC	POD	NE	DE	25.2	_	_
c.65A>G	Missense	2.34%	201442000	CM033926	DC	POD	NE	DE	25.2	21	_
c.65A>C	Missense	0.16%	201442000	CM033926	DC	POD	DE	DE	24.5	_	_
c.73C>T	Missense	2.15%	28936670	CM993125	DC	PRD	NE	DE	29.9	411	51
c.94G>A	Missense	7.69%	552617433	CM086531	DC	BENIGN	NE	TO	17.87	1	1
c.106C>A	Missense	0.37%	_	CM0910533	DC	PRD	NE	TO	23.5	_	_
c.126_142del	Indel	1.72%	_	CM109031	DC	PRD	DE	DE	34	_	_
c.133T>C	Missense	1.47%	779548360	_	DC	PRD	NE	TO	24.8	_	_
c.138C>G	Missense	1.72%	757461276	CM109031	DC	PRD	NE	DE	28.5	_	_
c.151T>C	Missense	1.47%	753937287	_	DC	POD	NE	TO	23.4	1	_
c.160G>A	Missense	0.37%	_	CM1110284	DC	BENIGN	NE	TO	22.7	_	_
c.175C>G	Missense	2.20%	387906775	CM108740	DC	PRD	NE	TO	18.81	_	_
c.202G>A	Missense	7.69%	_	_	DC	BENIGN	NE	TO	16.42	_	_
c.206T>C	Missense	1.47%	_	_	DC	PRD	NE	TO	23.1	_	_
c.230C>T	Missense	1.47%	_	_	DC	BENIGN	NE	TO	12.85	_	_
c.262delG	Indel	5.5%	606231360	CD051763	DC	POD	NE	TO	25.1	_	_
c.312delG	Indel	16.66%	774878026	CD067184	DC	BENIGN	DE	DE	25	_	_
c.325G>T	Nonsense	62.5%	_	CM082970	DC	NA	NA	NA	36	_	_
c. 326A>T	Missense	0.71%	_	CM110809	DC	BENIGN	DE	TO	10.39	_	_
c.340T>A	Missense	7.35%	_	_	DC	BENIGN	DE	TO	13.12	_	_
c.340T>C	Missense	32.35%	_	_	DC	BENIGN	DE	TO	18.11	_	_
c.353A>G	Missense	7.35%	_	_	DC	BENIGN	NE	TO	17.89	_	_
c.355G>T	Missense	1.02%	137852684	CM061152	DC	BENIGN	NE	TO	10.02	101	3
c.365T>C	Missense	0.30%	_	CM123330	DC	BENIGN	NE	TO	15.81	_	_
c.371A>G	Missense	8.82%	_	_	DC	BENIGN	NE	TO	11.83	_	_
c.377A>T	Missense	16.17%	_	_	DC	BENIGN	DE	TO	13.84	_	_
c.380C>A	Missense	0.16%	387906774	CM033928	DC	BENIGN	NE	TO	0.002	_	_
c.397C>T	Missense	8.82%	_	_	DC	BENIGN	DE	DE	19.71	_	_
c.397-400del	Indel	33.33%	_	_	DC	PRD	DE	DE	32	_	_
c.403G>A	Missense	13.23%	_	_	DC	BENIGN	NE	TO	16.4	_	_
c.403delG	Indel	5.94%	_	_	DC	BENIGN	NE	TO	26.4	_	_
c. 424C>T	Missense	26%	_	CM021252	DC	PRD	DE	DE	34	_	_
c. 431T>C	Missense	16.17%	747932354	_	DC	PRD	DE	DE	26.9	_	_
c.434T>C	Missense	0.74%	72554027	CM127833	DC	PRD	DE	DE	28.2	_	_
c.437C>G	Missense	0.38%	397516909	_	DC	PRD	DE	DE	31	_	_
c. 445C>T	Nonesense	6.47%	_	CM993126	DC	NA	NA	NA	37	_	_
c.448G>A	Missense	14.28%	201582515	CM098203	DC	PRD	NE	DE	29.1	_	1
c.461A>G	Missense	0.93%	587782928	_	DC	PRD	DE	DE	27.6	_	_
c.479A>C	Missense	50%	199475601	CM071903	DC	PRD	DE	DE	26.7	_	_
c. 482G>C	Missense	1.41%	137852685	CM061151	DC	PRD	DE	DE	33	1	1
c.498- 499ins C	Indel	6.25%	_	CI050501	DC	PRD	DE	DE	35	_	_
c.508C>T	Nonesense	12.12%	104893901	CM980448	DC	NA	NA	NA	37	_	_
c.512T>C	Missense	18.75%	_	CM044907	DC	PRD	DE	DE	27.9	_	_
c.512InsGC	Indel	37.5%	_	CI107550	DC	PRD	DE	DE	34	_	_
c.533C>T	Missense	7.61%	104893900	CM980449	DC	PRD	DE	DE	29.3	_	_
c.536C>T	Missense	2.21%	_	CM0910532	DC	PRD	DE	DE	27.7	_	_
c.538A>G	Missense	1.36%	_	_	DC	PRD	DE	DE	25.6	_	_
c.541C>T	Nonsense	0.3%	_	_	DC	NA	NA	NA	38	_	_
c.543G>C	Missense	36.36%	72554028	CM096566	DC	PRD	DE	DE	27.8	_	_
c.547A>G	Missense	42.64%	137852686	_	DC	POD	DE	DE	26	_	_
c.550A>T	Missense	4.34%	_	_	DC	POD	DE	DE	27.8	_	_
c.552 C>G	Missense	0.81%	_	CM135608	DC	POD	DE	DE	24.8	_	_
c.554G>T	Missense	18.75%	797045792	CM050301	DC	POD	DE	DE	30	_	_
c.557T>C	Missense	1.47%	_	_	DC	BENIGN	DE	DE	24.4	_	_
c.559C>T	Nonsense	8.82%	_	_	DC	NA	NA	NA	37	_	_
c.561G>C	Missense	4%	_	CM021253	DC	POD	DE	DE	25.8	_	_
c.564C>A	Missense	4.23%	_	CM993127	DC	PRD	DE	DE	26.3	_	_
c.565C>G	Missense	5.08%	_	CM993128	DC	POD	DE	DE	26.9	_	_
c.568C > T	Missense	5.5%	104893906	CM051567	DC	PRD	DE	DE	32	_	_
c.569G>T	Missense	1.65%	_	CM107631	DC	POD	DE	DE	34	_	_
c.569G>A	Missense	2.95%	_	CM044906	DC	POD	DE	DE	34	_	_
c.572A>G	Missense	1.69%	_	CM993129	DC	PRD	DE	DE	25.5	_	_
c.574A>T	Nonsense	3.52%	_	_	DC	NA	NA	NA	37	_	_
c.575A>C	Missense	8.82%	_	_	DC	PRD	DE	DE	26.9	_	_
c.575A>G	Missense	2.94%	_	_	DC	PRD	DE	DE	26	_	_
c.581A>G	Missense	2.94%	_	_	DC	PRD	DE	DE	25.9	_	_
c.592C>T	Nonsense	3.81%	104893903	CM980450	DC	NA	NA	NA	37	_	_
c.605- 606delTG	Indel	18.75%	_	CD050462	DC	POD	DE	DE	35	_	_
c.608A>G	Missense	0.66%	771533553	CM109892	DC	BENIGN	DE	DE	24.1	4	_
c.614T>A	Missense	8.82%	_	_	DC	BENIGN	NE	TO	22.9	_	_
c.615 delG	Indel	0.93%	_	_	DC	POD	NE	TO	33	_	_
c. 626C>T	Missense	0.71%	_	CM110810	DC	BENIGN	NE	TO	21.9	_	_
c.632C>T	Missense	4%	3729754	_	DC	PRD	DE	TO	23.4	21	1
c.646C>T	Missense	5.14%	104893905	CM013526	DC	PRD	DE	TO	33	_	_
c.656C>T	Missense	0.16%	104893902	CM013527	DC	PRD	DE	TO	33	_	_
c.676G>A	Missense	2%	_	CM1010049	DC	BENIGN	DE	DE	25.4	1	_
c.694G>A	Missense	0.3%	_	CM123331	DC	PRD	DE	DE	28.7	_	_
c. 701 InsTCCCT	Indel	0.88%	_	CI034942	DC	BENIGN	NE	TO	34	_	_
c.707C>A	Missense	0.3%	397515399	CM123618	DC	POD	DE	DE	24.7	1	_
c.721-728del	Indel	0.93%	587782930	_	DC	BENIGN	NE	TO	35	_	_
c.738T>A	Missense	22.7%	_	_	DC	POD	DE	DE	24.2	_	_
c.742T>C	Missense	7.35%	_	_	DC	POD	DE	DE	26.2	_	_
c.762delC	Indel	0.82%	_	CD107632	DC	BENIGN	NE	TO	28	_	_
c.768T>A	Missense	14.28%	104893907	CM066150	DC	NA	NA	NA	35	_	_
c.769C>G	Missense	3.33%	387906776	_ CM104914	DC	POD	NE	TO	8.926	_	_
c.777C>G	Nonsense	6.77%	_	CM993130	DC	NA	NA	NA	36	_	_
c.792C>A	Nonsense	0.91%	_	CM023353	DC	NA	NA	NA	37	_	_
c.795C>A	Missense	37.5%	_	CM113773	DC	POD	NE	DE	25.7	_	_
c.809G>A	Missense	0.93%	587782931	_	DC	POD	DE	DE	25.3	7	_
c.823C>A	Missense	0.16%	368366482	CM033929	DC	POD	NE	DE	23.7	10	3
c.848C>A	Missense	0.74%	375086983	CM108634	DC	BENIGN	NE	TO	11.39	7	_
c.delAAC871	Indel	0.16%	756974215	_	DC	NA	NE	TO	16.05	_	_
c.872A>T	Missense	1%	756974215	_	DC	POD	NE	DE	27.1	_	_
c.877delG	Indel	26.6%	749577978	_	DC	NA	NA	NA	35	_	_
c.880A>C	Missense	20.58%	_	_	DC	BENIGN	NE	DE	23.1	_	_
c.896A>G	Missense	52.94%	137852683	_	DC	BENIGN	NE	TO	17.59	_	_
c.958G>A	Missense	25%	_	_	DC	PRD	DE	TO	25	_	_
c.965G>A	Missense	2.94%	376426882	_	DC	POD	DE	DE	33	_	_
c.967G>A	Missense	0.16%	_	CM033930	DC	POD	DE	TO	24.9	_	_

Polyphen-2, score =0-0.15: Benign; score =0.15-0.85: Possibly damaging; score =0.85-1: Probably damaging; PROVEAN, score ≤ -2.5: Deleterious; score >-2.5: Natural; SIFT, score ≤ 0.05: Deleterious; score >0.05: Tolerable; CADD, Phred ≤ 20: Damaging; Phred >20: Natural. DC: Disease Causing; POD: Possibly Damaging; PRD: Probably Damaging; DE: Deleterious; NE: natural; TO: Tolerable; NA: Not Available.

### Prediction of the normal and mutant models

**Figure 4:**
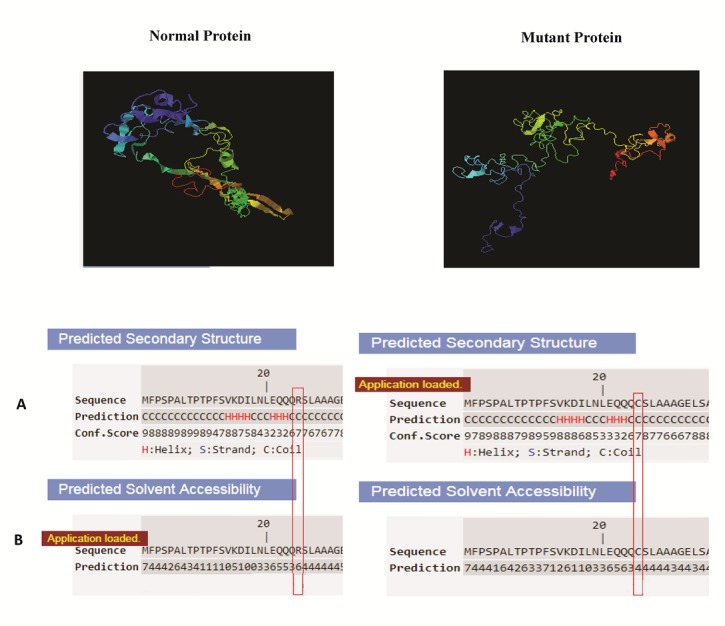
Three-dimensional models of normal (left) and mutant (right) Nkx2-5, p.R25C, proteins were predicted by I-TASSER (Iterative Threading Assembly Refinement). A: Representation of predicted Secondary Structure. B: Representation of predicted Solvent Accessibility.

Five structural/functional models of normal Nkx2-5 protein were obtained by I-TASSER as an output. We selected the structure with the highest scores, C-score: -4.50, TM-score: 0.25±0.07 and RMSD: 17.8±2.5Å. Moreover, we captured the three-dimensional models of mutant protein p.R25C generating by I-TASSER and selected the structure with the highest scores, C-score: -3.38, TM-score: 0.34±0.11 and RMSD: 14.6±3.7Å. The result assessing showed the solubility of mutant protein was reduced in comparison with normal protein but the protein structure was the same Indeed, solvent accessibility was predicted both native Arginine residue with score of 6 and variant Cysteine residue with score of 4 as buried exposed (Figure 4).

### Protein association analyses

**Figure 5:**
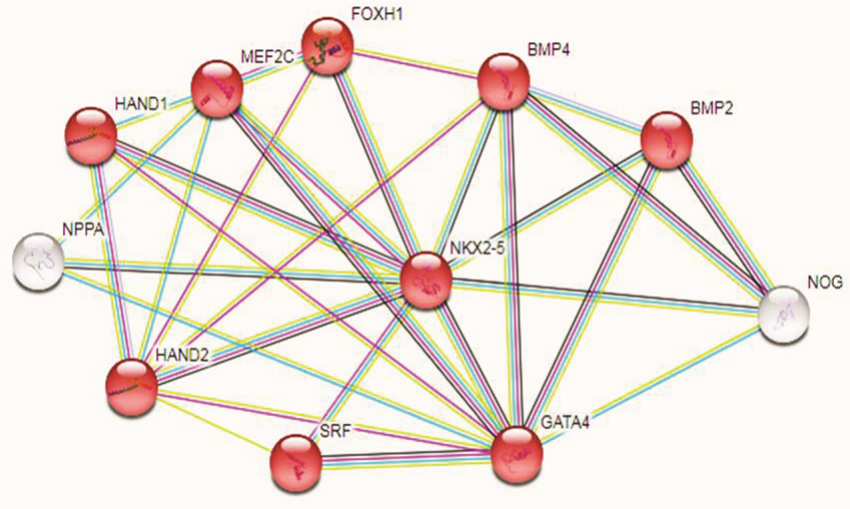
The Nkx2-5 interaction network. HAND1: Heart and neural crest derivatives expressed 1, HAND2: Heart and neural crest derivatives expressed 2, MEF2C: Myocyte enhancer factor 2C, GATA4: GATA binding protein 4, TBX5: T-box, 5SRF: Serum response factor, BMP4: Bone morphogenetic protein 4, MEF2A: Myocyte enhancer factor 2A, SMAD4: SMAD family member 4, NOG: noggin. This figure was captured from STRING site (V9.1)

Any change in Nkx2-5 pathway network can affect on this protein functions or vice versa. The STRING analysis represented 10 interactive proteins; GATA4, MEF2A, TBX5, SMAD4, HAND1, HAND2, MEF2C, BMP4, NOG and SRF for Nkx2-5 protein in protein-protein association software (Figure 5).

## Discussion

CHD is the most common birth defect in worldwide.^71^ Although there are several important genes which play important role in the CHD etiology, but the *Nkx2-5* is topping the list. *Nkx2-5* is one of the master transcription factors of heart development that regulates cardiac ion channels.^72,73^ This gene was identified as the first gene involved in CHD by genetic association studies in large families.^20,74^ We determined the frequency/distribution of *Nkx2-5* mutations and evaluated these mutations by using computational tools (Mutation Taster, SIFT, Polyphen2, PROVEAN and CADD).

This protein consists some conserved regions: DNA binding home domain (HD), peptide conserved TN-domain near the amino acid terminus and NK2-domain located c-terminal to the HD. Studies have demonstrated that HD domain has critical role in DNA binding, interaction with other proteins and transcriptional regulation.^75, 76^ In the majority of reported cases, the variant is a missense mutation (33 missense mutations) located within the HD domain of the *Nkx2-5* gene (Figure 2). Moreover, the most common CHD types resulting from *Nkx2-5* mutation were ASD and VSD.

In the past years, *in silico* analyses as an efficient tool has classified variants as being neutral or lethal.

Both SIFT and Polyphen2 are the most common *in silico* prediction tools applied in diagnostic laboratories. The approach which was used to classified variants as “Benign” or “Pathogenic” according to combined predictions from the five computational tools. This approach was applied to ensure all likely pathogenic variants of *Nkx2-5* gene would not be missed. In current study, we could determine high confidence information regarding the effect of amino acid change on *Nkx2-5* structure/function applying solely computational tools. The present work is the first attempt to asses all mutations of the *Nkx2-5* gene and overall, we reported 105 variants of *Nkx2-5* gene. Among them, c.380C>A variant was predicted to be benign by SIFT, Polyphen2 and PROVEAN tools but disease causing by Mutation taster. However, the low CADD score (Phred score= 0.002) confirmed that it can be a polymorphism. The c.541C>T had the highest CADD score (Phred score=38) and was only observed in England. Given this information, it can be deduced that c.541C>T is a mutation with founder’s effect which resulting in ASD in England. The highest frequency *Nkx2-5* mutations, about 99.6%, was revealed in Germany. It means that more association studies in this country can discover more new mutations in this gene.

Given that the c.73C>T (p.R25C) was distributed in all continents, it seems to be a hotspot position. Although HGMD documented this mutation as a pathogenic variant, but the 1000 Genome and ExAC databases reported it as a heterozygous form. Moreover, most of the software’s predicted it as a disease causing variant. c.325G>T and c.896A>G were observed with the high frequency just in Germany, this indicates a founder effect of these mutations. c.325G>T is a nonsense mutation which generates a truncated protein, while c.896A>G was predicted as a benign variant by several prediction tools, it was reported as a pathogenic variant in HGMD and not registered in 1000 G and ExAC.

Among Nkx2-5 network proteins, BMP2 protein is a cardiac factor which elicits expression ectopically of the heart markers GATA4 and Nkx2-5. It plays critical role in myocardial differentiation and regulation of proliferation. The Nkx2.5 has binding site in SMAD4 enhancer, thus BMP2 activity is needed for heart progenitor characteristics.^77^ TBX5 and Nkx2-5, both of them operate as co-activators for GATA4 pathway activity, any changes in these proteins can disrupt cardiac septation.^78^ This study indicates that the frequency and distribution of *Nkx2-5* mutations is more in Europe (Figure 3) and Asia and America are standing in next steps. This result shows that maybe the source of *Nkx2-5* mutations was from Europe and by migration transferred to other continents. Finally, it should be noted there are many modifier factors in *Nkx2-5* pathway which might affect on manifestation resulting from *Nkx2-5* mutations. Large population studies, variants frequency assessment in both of normal and patient population, functional study of mutations by animal models and evaluate expression level arising from mutations, can improve our understanding in this category.

## Conclusion

Here, *in silico* analyses and structural model of *Nkx2-5* have been submitted for the first time. *Nkx2-5* plays critical role in embryonic cardiac development and has several important mutable regions with high distribution. Our results indicated that *Nkx2-5* gene can be a significant candidate for CHD etiology investigation. Bioinformatics approaches permit large numbers of variants to be evaluated at the same time and predicted effects of all variants at the protein level. It should be noted that our information are according to database, however, any pathogenic variants should be experimentally confirmed.Regarding that some mutations observed only in Germany, also high frequency and diversity of mutations in this country, it seems *Nkx2-5* has a significant role in this part of the world.

## Competing interests

None.

## Ethical approval

The study is performed in accordance with the Helsinki Declaration and has been approved by the Rajaei Cardiovascular, Medical and Research Center (RHC.AC.IR.REC.1395.46; 24 December 2016) ethics Committees.

## Acknowledgments

This project was provided by Rajaie Cardiovascular Medical and Research Center, Tehran, Iran, Zanjan University of Medical Sciences, Zanjan, Iran and Islamic Azad University, Science and Research Branch, Tehran, Iran.
